# Machine Learning for the Design and the Simulation of Radiofrequency Magnetic Resonance Coils: Literature Review, Challenges, and Perspectives

**DOI:** 10.3390/s24061954

**Published:** 2024-03-19

**Authors:** Giulio Giovannetti, Nunzia Fontana, Alessandra Flori, Maria Filomena Santarelli, Mauro Tucci, Vincenzo Positano, Sami Barmada, Francesca Frijia

**Affiliations:** 1 Institute of Clinical Physiology, National Research Council (CNR), 56124 Pisa, Italy; mariafilomena.santarelli@cnr.it; 2 Department of Energy, Systems, Territory and Construction Engineering, University of Pisa, 56126 Pisa, Italy; nunzia.fontana@unipi.it (N.F.); sami.barmada@unipi.it (S.B.); 3 Bioengineering Unit, Fondazione Toscana G. Monasterio, 56124 Pisa, Italy; alessandra.flori@ftgm.it (A.F.); positano@ftgm.it (V.P.); f.frijia@ftgm.it (F.F.)

**Keywords:** magnetic resonance imaging, machine learning, RF coils, genetic algorithm

## Abstract

Radiofrequency (RF) coils for magnetic resonance imaging (MRI) applications serve to generate RF fields to excite the nuclei in the sample (transmit coil) and to pick up the RF signals emitted by the nuclei (receive coil). For the purpose of optimizing the image quality, the performance of RF coils has to be maximized. In particular, the transmit coil has to provide a homogeneous RF magnetic field, while the receive coil has to provide the highest signal-to-noise ratio (SNR). Thus, particular attention must be paid to the coil simulation and design phases, which can be performed with different computer simulation techniques. Being largely used in many sectors of engineering and sciences, machine learning (ML) is a promising method among the different emerging strategies for coil simulation and design. Starting from the applications of ML algorithms in MRI and a short description of the RF coil’s performance parameters, this narrative review describes the applications of such techniques for the simulation and design of RF coils for MRI, by including deep learning (DL) and ML-based algorithms for solving electromagnetic problems.

## 1. Introduction

Magnetic resonance imaging (MRI), a noninvasive diagnostic technique based on the phenomenon of nuclear magnetic resonance (NMR), can be considered one of the most important medical imaging approaches for the diagnosis and follow-up of diseases that affect different tissues and organs. MRI requires a static magnetic field, B_0_, associated with gradients and radiofrequency (RF) pulses for image production, the latter with a B_1_ RF field frequency in the range of 21–298 MHz, corresponding to B_0_ field strengths from 0.5 to 7 T for proton MR experiments. The B_1_ field is generated and picked up by transmit and receive RF coils, respectively [1].

The transmit RF coil needs to produce a highly uniform magnetic field in the desired field of view (FOV), since the extension of the region being analyzed is not known a priori; therefore, they are usually large in order to optimize the magnetic field’s homogeneity and include a significant tissue volume.

The function of the receive RF coil is to maximize signal detection while minimizing noise, and for this purpose, it is optimal to minimize the coil dimensions.

In general, both transmit and receive RF coils have to be adapted to the specific application and to the human body portion dimension, although they also need to provide good performances with slightly different subjects. To optimize an RF coils’ performance for a given application and avoid the time-consuming and expensive trial-and-error approach, an accurate design and simulation process must be followed, including inductance, conductor losses, and magnetic field pattern calculation, which can be performed with recent computer simulation techniques. Historically, coils simulation was performed by using two different methods. The first one (magnetostatic approach) provides an efficient formulation to estimate coil performance in a fast and practical way and is mainly employed for the design of low-frequency-tuned coils. The second approach, based on full-wave methods, is able to model the interaction between the RF field and the human body, and, therefore, is useful for the simulation and design of coils in a loaded condition and tuned at higher frequencies [1].

Among the various emerging strategies, machine learning (ML) is worthy of mention, as it is largely used in many sectors of engineering and sciences, and such ML algorithms, optimized for solving electromagnetic problems, are well-suited for the design of RF coils for MRI. Machine learning is a subfield of artificial intelligence (AI) focused on the concept of “learning from data”. ML algorithms are not explicitly programmed. Instead, a generalizable model is designed, defined by several parameters (hyperparameters) that are “fitted” to data to optimize the model. ML algorithms can be divided into unsupervised and supervised algorithms. Unsupervised algorithms work on unlabeled data, autonomously finding patterns or relationships within the data. Clustering is the most commonly used unsupervised ML algorithm. Evolutionary computation, including genetic algorithms, is also a part of unsupervised ML. Supervised algorithms are trained on labeled data, where each input is paired with the correct output (ground truth). The objective is to learn to map from inputs to outputs so the model can make predictions on new, unseen data. Neural networks are typical examples of supervised ML algorithms. Machine learning is widely applied in the magnetic resonance (MR) field, with applications in image analysis, reconstruction, and interpretation [2]. ML methods, particularly convolutional neural networks (CNNs), have shown success in automating the segmentation of anatomical structures and abnormalities in MR images. Segmentation models aid in tasks such as tumor delineation, organ delineation, and lesion detection, enhancing the efficiency of clinical diagnosis. The topic has been well covered by recent reviews [3,4,5]. ML algorithms are also increasingly used for the automated detection and classification of diseases from MR images. This includes the identification of neurological disorders, musculoskeletal conditions, and oncological abnormalities. Deep learning models, including CNNs and recurrent neural networks (RNNs), demonstrate promising results in distinguishing between normal and pathological conditions [6,7]. Another field of application of ML is that regarding motion correction techniques, which are able to correct motion artifacts in MR imaging [8,9]. ML is also used for the quantitative analysis of MR images, providing precise measurements of tissue properties and characteristics [10]. This is valuable for applications such as perfusion imaging, diffusion-weighted imaging, and spectroscopy [11]. Finally, generative models, including variational autoencoders (VAEs), are used for data augmentation, enabling the creation of realistic synthetic MR images. This aids in training robust and generalizable ML models with limited labeled data [12,13].

ML techniques have also been extensively applied to MR image reconstruction, a field strictly linked with radiofrequency MR coils, as image reconstruction algorithms are dependent on the coil type and geometry in addition to the sampling method of the acquired data. ML could also be applied to RF coil design as a means to reduce the drawbacks linked to the computational complexity of EM simulations. ML-based optimization algorithms, such as genetic algorithms, could be used to find “unexpected” coil geometries that provide the desired magnetic field distribution. In principle, physically informed neural networks could provide very fast EM simulations of RF coil fields, allowing for the exploration of a wider range of geometries with respect to traditional methods.

This narrative review describes the applications of ML algorithms for the design of RF coils for MRI. To our knowledge, a review on this topic has not been previously reported.

The paper is organized as follows: After a description of the ML for image reconstruction, which is a process closely linked to the geometry and typology of RF coils (Section 2), the different RF coil categories and their performances in terms of losses and magnetic field homogeneity are described (Section 3), with a particular focus on the description of the constraints for designing an optimal RF coil. Moreover, an overview of the deep learning (DL) and ML algorithms for solving electromagnetic problems is included (Section 4), which is useful for the design of RF coils for MRI, being schematizable as antennas working in the near-field region. Finally, Section 5 summarizes the papers that have employed ML algorithms for the design and simulation of RF coils, mainly based on genetic algorithms (GA).

## 2. Machine Learning Applications in MR Reconstruction

MR image reconstruction is a process that transforms the acquired k-space signal, which is corrupted by measurement noise, into the final MR image. For a fulfilled k-space, the transformation is straightforward and effectively approximates the Fourier transform. The possibility of using a deep neural network for the reconstruction of a fulfilled k-space was demonstrated by Zhu et al. [14], who obtained a significant reduction in the needed processing time. However, in several MR applications, the k-space is undersampled to speed up the image acquisition process. These techniques help in obtaining high-quality images from undersampled data, reducing scan times significantly. Two main approaches for image reconstruction from undersampled data can be recognized: parallel imaging and compressed sensing. In parallel imaging, multi-channel receiver arrays are used to compensate for the undersampling of k-space. In fact, receiver coils exhibit spatially varying responses, which can be weighted to unfold aliased images or estimate missing k-space samples. The two main parallel imaging techniques, SENSE (sensitivity encoding) and GRAPPA (generalized autocalibrating partial parallel acquisition), are routinely used in the clinical environment. Compressed sensing (CS) is a general signal analysis technique able to reconstruct a subsampled signal that is sparse (i.e., contains few non-zero elements compared to its size) in some domains. Although MRI images are not typically sparse, they contain many redundancies and can be represented as sparse in other domains, such as wavelet or finite difference.

**Figure 1:**
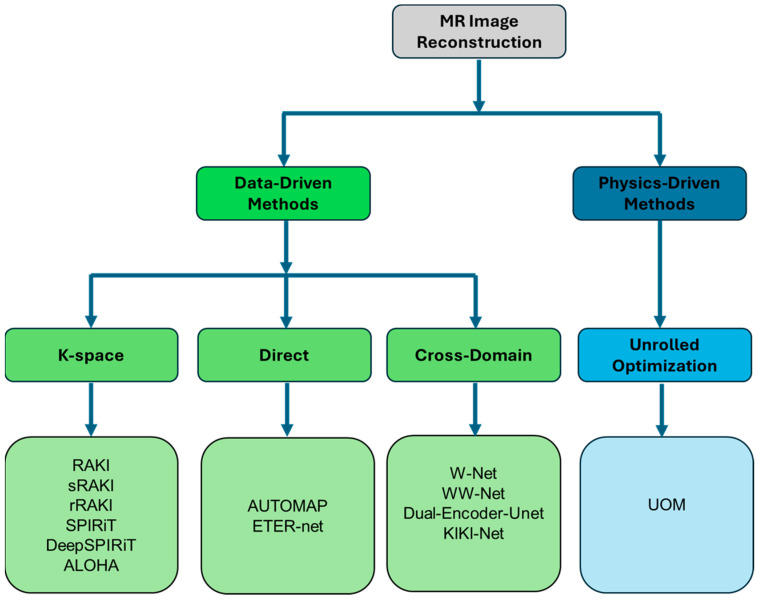
Diagram representing the different types of ML methods used in MR image reconstruction.

Many ML algorithms have been developed to improve MRI image reconstruction from undersampled k-space [15,16,17,18], following different approaches that can be classified into data-driven and physics-driven methods (Figure 1):

### 2.1. K-Space Methods

In k-space method approach, the ML algorithm operates at the k-space level, transforming the undersampled k-space into a fulfilled k-space. Then, classical FFT transformation is employed for image reconstruction from the k-space.

Among k-space methods, supervised k-space enhancement, mimicking GRAPPA, can be performed using large training databases without the need for explicit coil sensitivity information or exploiting a small amount of fully sampled reference data, known as the auto-calibration signal (ACS). Akçakaya et al. [19] trained a convolutional neural network (CNN) to obtain a non-linear estimation of missing k-space lines (RAKI method) that outperformed the conventional linear k-space interpolation-based methods. RAKI is trained on ACS data so as not to require a large training database, but is trained using the ACS data from each scan. The method was extended to arbitrary sampling patterns by self-consistent RAKI (sRAKI) [20]. Further advances include residual RAKI (rRAKI) [21] and LORAKI [22], which simultaneously include magnitude, phase, and parallel imaging data.

The RAKI approach, designed for uniform undersampling patterns, was extended to arbitrary sampling using the SPIRiT method [23]. A different approach (DeepSPIRiT) uses CNNs trained on large databases for k-space interpolation [24]. DeepSPIRiT has the potential to reduce acquisition time, as it does not require calibration data for a given scan. In the study, the ALOHA method (a Hankel matrix-based approach) was replaced with a CNN. This method improved both the computational time and the reconstruction quality compared to the original ALOHA [25]. Generative adversarial networks (GANs) and deep learning approaches are commonly used to fill in missing k-space data and reconstruct images with improved spatial and temporal resolution. ML-based image denoising can be employed to avoid the generation of incoherent, noise-like aliasing in under-sampled MR reconstruction, working on the k-space [26,27,28,29].

### 2.2. Direct Methods

Direct methods learn the transform between the undersampled k-space and the image domains. Several direct methods for the direct reconstruction of the MR image from the undersampled k-space were proposed, such as AUTOMAP (automated transform by manifold approximation) [14]. AUTOMAP was trained on synthetic undersampled k-space data and the respective reconstructed images. The computational complexity of AUTOMAP can be reduced by using a multi-layer perceptron network instead of the deep learning network used by AUTOMAP. In the ETER-net (end-to-end MR image reconstruction using recurrent neural network) approach, a bidirectional RNN was used for the direct reconstruction of high-resolution images [30].

### 2.3. Cross-Domain Methods

Cross-domain methods operate in both k-space and image domains. The underlying assumption is that, as ML elaborations of k-space and images have different characteristics, a combination of them might improve the overall performance with respect to sequential elaborations.

Cross-domain method architectures typically employ a frequency domain subnetwork for the estimation of k-space missed samples and an image domain subnetwork for removing residual artefacts. The U-Net architecture is often used, as in W-Net [31] and in the multi-domain CNN proposed by El-Rewaidy et al. [32]. Dual-Encoder-Unet, where a single U-Net with two encoders that operate simultaneously on both k-space and image domains, is also used [33]. The W-Net approach was extended to WW-Net, employing a cascade of U-Net networks [34]. An important finding of this study is that cross-domain networks perform better in multi-channel coil settings, where the inter-coil correlations can be efficiently exploited by k-space domain networks. Other cascade-based approaches have been proposed, such as KIKI-Net [35].

### 2.4. Unrolled Optimization Methods (UOM)

Unrolled optimization methods follow the same approach of iterative optimization algorithms used in CS. Iterations are “distributed” on a neural network, achieving the transformation of the k-space to the reconstructed image.

All of these approaches are purely data-driven, as networks basically learn from image pairs (e.g., k-space and reconstructed image) that should be representative of the knowledge domain. The information on the underlying acquisition physics is entirely discarded. Hence, k-space and MR image consistency cannot be guaranteed. To obtain consistent results on all the spectra of MR scanners/configurations, the training data should be representative of a very large number of acquisition parameters. For instance, all possible MR coil configurations in terms of coil number, shape, and geometrical disposition should be present in the training data. Due to the difficulty of obtaining these data, data-driven models are often “specialized” on particular scanner configurations used to acquire training data.

To solve these issues, physics-driven methods, which explicitly use the known physics-based forward imaging models in deep learning architectures and/or training, have been proposed. These methods have gained popularity in the MRI community due to their incorporation of MR domain knowledge [36].

**Figure 2:**
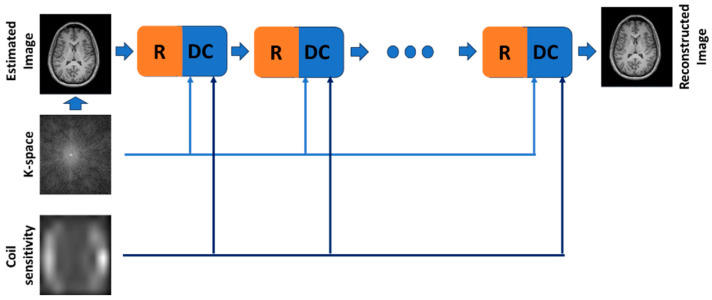
Workflow of the unrolled optimization methods (UOM), the most used deep-learning, physics-based method for MR image reconstruction.

The most well known physics-based methods are the unrolled optimization methods (UOM), which hold several advantages. UOMs do not need manual tuning, which is a long process, and image reconstruction is fast because UOMs are trained to produce results with few iterations. In classical computational MR, for undersampled k-space, image reconstruction is obtained by solving an inverse problem, incorporating additional domain knowledge as redundancies among coils and minimizing an appropriate objective function. The minimization of the objective function is typically obtained by an iterative algorithm using an appropriate regularizer. The conventional iterative approach can be “unrolled” using UOM techniques and solved for a fixed number of iterations. The algorithm’s parameters, such as regularization parameters and update rate, can be treated as hyperparameters and fitted using a training dataset. Typically, a UOM method minimizes the sum of two terms: a data consistency term (DC) dependent on the current solution x and the labeled data y and a regularization term (R) that depends on x only (Figure 2). K-space and coil sensitivity data, if available, are provided to the DC block that minimizes the difference between the output image and the reference image. The R block is typically constituted by a CNN network that learns the optimal regularization parameters. The number of R/DC pairs is typically small to speed up the image reconstruction process.

The minimization operation performed by DC blocks can be performed via different optimization algorithms, and the R blocks can be implemented using different CNN architectures. These aspects characterize the different UOM implementations. A comprehensive list of UOM implementations can be found in [15].

## 3. Theory and Optimization of Coil Design

According to their shapes, RF coils can be categorized into volume, surface, and phased-array coils [37].

Volume RF coils, mainly constituted by Helmholtz and birdcage coils, are often employed both as transmit and receive RF coils since they are able to generate a uniform field in a large region surrounding the human body portion. The “filling factor” gives a measure of the geometric relationship between the volume RF coil and the object to be imaged. In particular, the filling factor can be maximized by designing coils whose cross sections mimic human anatomy [38], although fitting the coil close to the object can potentially decrease patient comfort.

Surface RF coils, constituted by loops of various shapes (circular, rectangular, elliptical), are much smaller than the volume coils since they have to provide high SNR images, even if with relatively poor magnetic field homogeneity. To have such good SNR in the region of interest (ROI), the size and geometry of the RF coil should be adapted to the depth of the sample under study [39]. For specific applications, such as in vertical B_0_ MRI systems, surface RF coils with butterfly and figure-of-eight geometry producing transverse RF fields can be designed [40]. Finally, phased-array RF coils [41], whose single elements are constituted by loops of different geometries, are able to achieve good SNR values typical of surface RF coils, with large sensitivity regions, which are usually obtained with volume RF coils. The optimal design of phased-array RF coils has to be taken into account for the minimization of the mutual inductance between the coil elements in order to avoid SNR losses due to coil resonant frequency modification. In particular, adjacent coil elements have to be overlapped to yield zero mutual inductance, while the reduction in the interference between the overlapped elements and the others in the array can be performed by connecting each coil element to a low-input impedance preamplifier [42]. Successively, an optimal image reconstruction algorithm must be used to combine the individual coil images into a single composite image with full FOV [43]. Phased-array RF coils are also employed in parallel imaging applications [44], where the magnetic field spatial variation in the single coil elements permits the spatial encoding of the signal, which provides substantial reductions in image acquisition time.

In RF coils, the current which flows in the conductor has to be maximal at the Larmor frequency (*f*_0_ = *γ*/2*π B*_0_, where *γ* is the gyromagnetic ratio with 42.58 MHz/T value for ^1^H nucleus). This frequency corresponds to the coil resonant frequency and can be calculated as:

(1) $$f_{0}=\frac{1}{2\pi \sqrt{LC}}$$

where inductance *L* takes into account the energy stored in the magnetic field and capacitance *C* mainly results from the contribution of the discrete capacitors. The current which flows in the coil conductor is mainly limited by loss mechanisms (schematized with a *R* total resistance), which take into account the conductor losses (*R_coil_*); sample losses (*R_sample_*); and tuning capacitor, soldering, and radiative losses (*R_extra_*). Such an RLC circuit is characterized by a *Q* quality factor [1]:

(2) $$Q=\frac{2\pi f_{0}L}{R}=\frac{1}{R}\sqrt{\frac{L}{C}}$$

and by the ratio *r* between the unloaded coil quality factor (*Q_unloaded_*) and that with the coil loaded with the human body portion or a phantom which mimics it (*Q_loaded_*):

(3) $$r=\frac{Q_{unloaded}}{Q_{loaded}}=1+\frac{R_{sample}}{R_{coil}+R_{extra}}$$

Another parameter which characterizes coil performance is the sensitivity, defined as the *B*_1_ magnetic field induced by the RF coil at a given point per unit of supplied power *P*, as follows [39]:

(4) $$\eta =\frac{B_{1}}{\sqrt{P}}$$

The maximization of the coil sensitivity will also maximize the SNR.

The coil inductance *L* should be minimized in order to lead to a lower conservative electric field in the sample for the same magnetic field amplitude. Such an electric field, generated by the scalar potential existing near the coil conductors, does not contribute to the magnetic resonance process and can lead to sample heating [45]. The electric field can also be reduced by segmenting the coil with capacitors, although the addition of many capacitors and the soldering connections may reduce the coil’s Q factor [46].

As well as having a sufficiently high breakdown voltage, the capacitors *C* must have a high-quality factor in order to minimize the electrode resistance and dielectric losses [47]. Moreover, in the choice of capacitors, it must be taken into account that their quality factors depend on the frequency [48].

Regarding a coil’s total resistance *R* and its different contributions, it is important to underline that an efficient coil should be designed by minimizing the coil noise (*R_coil_*) with respect to the sample noise (*R_sample_*), because coil conductor resistance affects data quality by reducing the SNR, especially for coils tuned to a low frequency [49].

In dependence on their cross-sectional shape, conductors used for RF coil construction can be categorized into strips (rectangular shapes, characterized by width *w* and thickness *t*) and wires (cylindrical rod shapes, defined by their radius *a*). Due to the “lateral skin effect” [50], the current distribution of strip conductors is less uniform than that of the wire ones, and such behavior guarantees better performance in terms of quality factors for coils constituted by wire conductors [51]. However, wire conductors are difficult to handle for coil building, as they require qualified mechanical personnel.

Another strategy for minimizing the conductor losses is to employ Litz wires, conductors constituted by multiple individually insulated strands twisted or woven together and arranged to occupy all the positions within the conductor. Such configurations provide an equal distribution of the flowing current among the separated strands and a consequent reduction in coil losses [52]. Finally, conductor losses can be reduced by lowering the coil temperature, which is achieved by cooling the conductor material [53] or by using cryogenically cooled high-temperature superconductors [54].

Radiative losses occur when conductor lengths are in the range of a wavelength, and so must be taken into account for large coils tuned at very high frequencies. To avoid a coil acting as a bent dipole and its current distribution becoming no longer uniform, the coil conductor can be segmented so that each coil segment is a small fraction (<1/20) of the wavelength [37].

The B_1_ magnetic field homogeneity is an important parameter in coil simulation and design, because it strongly affects the FOV. The estimation of the coil’s magnetic field pattern can be performed by using two different approaches. The first method, which is based on magnetostatic theory and, therefore, is useful for the design of low-frequency-tuned coils, implies the assumption of a nearly static field, which is valid for coils whose size is much lower than the wavelength. When the coil tuning frequency increases, RF fields interact more strongly with the human body, and different full-wave methods based on solutions to Maxwell’s equations toned to be employed for field calculation, including the finite-difference time-domain (FDTD) method [55], the finite element method (FEM) [56], and the method of moments (MoM) [57], although the computation times of such full-wave methods are much longer compared to the magnetostatic approach [58,59].

For optimizing MR experiments, quadrature coils producing/receiving circular polarized magnetic fields need to be designed in order to reduce the power requirement in transmit by a factor of two and to increase the received signal SNR by a factor of $\sqrt{2}$ [60]. This quadrature coil design must be performed by minimizing the mutual coupling between coil channels.

In some applications, imaging and multi-nuclear spectroscopy have to be integrated, and the magnetic resonance system must be able to operate at two different frequencies. The ideal hardware setup would require the use of a single dual-tuned coil operating at ^1^H and X nucleus frequencies, guaranteeing data acquisition in sequence without disturbing or repositioning the patient. However, an optimal coil design should guarantee the minimization of interactions between the X nucleus and the proton signals, starting from the geometrical decoupling of the channels [37].

In the coil design, another issue has to be taken into account. The interaction of the B_1_ field with gradient and shim coils can degrade the RF coil’s performance in terms of the SNR, while spurious resonant frequencies can be generated. Such an interaction can be minimized by using a passive shielding system, constituted by sheets of ferromagnetic material with high-permeability or high-conductivity material [61]. However, in the coil simulation and design phases, it must be taken into account that the shield strongly modifies resonance frequencies and field distribution in the RF coil [62].

## 4. Deep Learning and Machine Learning Applied to Electromagnetic Problems

The design of radiofrequency coils for MR applications is closely connected, as described earlier, to the analysis of a uniform magnetic field distribution in a specific region of interest (ROI) at a desired frequency. Indeed, magnetic resonance coils are structures typically made resonant through the use of tuning capacitors. The operating frequencies of RF coils for MRI are such that the dimensions of the magnetic resonance coil are much smaller than the corresponding wavelength of interest. Therefore, in the design of such systems, one can resort to the steady-state approximation without committing substantial errors in the calculation procedure of field distributions in the vicinity of the coil itself. Furthermore, since a uniform field is required in the regions immediately surrounding the coils, one can certainly speak of the near field, where any radiative phenomena can be neglected.

The approach of utilizing machine learning algorithms for solving electromagnetic problems, which can be divided into so-called forward problems and inverse problems, is well-suited for the design of RF coils for magnetic resonance, given that for both the forward and inverse solutions, employing deep learning provides a notable advantage—the ability to directly manipulate images. This efficiently represents various elements, such as geometry (boundaries, material interfaces), sources, and results (e.g., field distributions presented as color maps), through image-based representations. ML algorithms can perform effectively in such scenarios.

Over the past few years, extensive research efforts have been directed towards employing neural network (NN) methodologies, particularly in the form of DL models, to address electromagnetic (EM) field problems.

**Figure 3:**
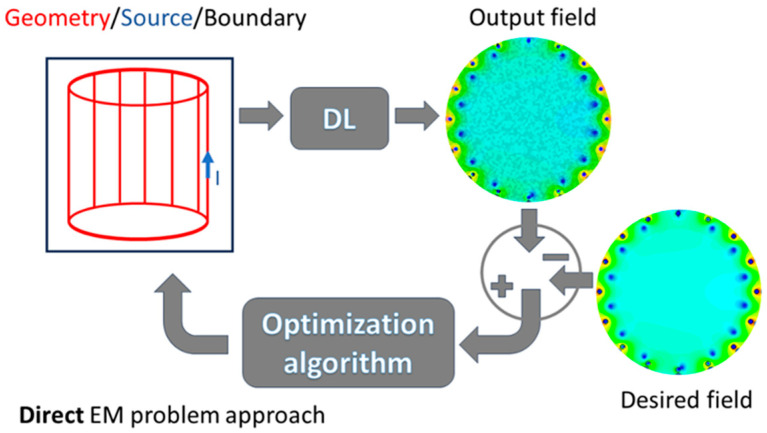
Direct/forward EM problem typically solved in the literature through deep learning paradigms.

Neural networks (NNs), often configured as DL models, have found an application in solving forward EM problems. In general, properly trained NNs can produce the “expected” output when a set of quantities is given as the input. When the input is a set of geometrical parameters and source quantities, and the output is one (or more) electromagnetic quantity, the NN is capable of solving the direct problem. It is known that the generalization capabilities are one of the key aspects to be taken into account when dealing with NNs, i.e., the NN will produce a good approximation of the real output field when it has been trained with a dataset that is not “far” from the new input–output cases on which it is expected to work. In other words, the NN serves as a surrogate model of the physical phenomenon, usually modeled by a numerical method. The complexity of the NN increases with the number of inputs when complex geometry is considered, and it cannot be described by a set of parameters (in electrical engineering, the most common case is the reluctance motors). The DL paradigm is used, and the input to the network is an image describing the geometry and the source. Again, the result is a surrogate model capable of producing an accurate output when properly trained (Figure 3). This has been exploited when repeated calculations of the same quantity are needed, with “non-dramatic” variations in the geometry, something that is typical in topology optimization procedures. The studies cited in [63,64,65,66,67] are characterized by the use of DL models for the solutions to EM problems and the optimization of EM devices. The primary advantage, as previously explained, lies in the ability to achieve accurate evaluations of the desired quantities in a minimal amount of time (if compared to a numerical method). This efficacy is attained through appropriate training of the NN using a well-suited dataset. However, it is essential to acknowledge that the cost associated with generating the training dataset and building the model for dataset calculation is non-negligible and must be factored into the overall assessment of DL’s capability to tackle EM problems. In [68], a recent review on the forward EM problems solved by relying on NNs and DL approaches is presented.

A more recent application of DL involves training NNs to address EM inverse problems, such as identifying the magnitude and/or geometrical characteristics of the source when the field distribution is known at specific points [69,70,71].

Theoretically speaking, forward EM problems are characterized by a unique solution when sources and boundary conditions are properly defined. This solution can be obtained using a proper numerical solution with a certain level of approximation. In contrast, inverse problems are commonly addressed by minimizing a reconstruction error, often incorporating a regularization technique; this is essential, as the raw observed data typically give rise to non-unique and ill-conditioned solutions. A common application is the calculation of the sources (identified by source position and/or magnitude) starting from the measured data: the simplest example could be the calculation of the positions of conductors carrying a specific current from the magnetic field measurement through a set of points in space.

**Figure 4:**
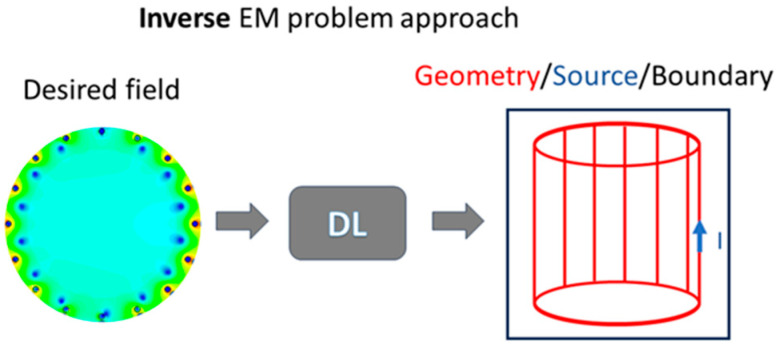
Inverse EM problem solved in the literature through deep learning paradigms.

The use of DL for inversion, in this context, can be viewed as a regularization procedure. In more detail, the training set is generated as a direct problem, but the NN is trained by conceptually switching the inputs and the outputs; in the example described above, the field, at several points, is calculated given a specific source distribution. In this way, a direct problem dataset is generated, but the network is trained using the magnetic fields as input and the source parameters as output. The final result is an NN (or DL network) performing the inversion (Figure 4). Several authors have explored these aspects in the literature, particularly in applications related to image processing [72,73,74,75].

In [76], a methodical examination of how various learning parameters influence the (re-)construction of an electromagnetic inverse problem (EIP) model is presented, contrasting it with more established regularization techniques. The authors use a magneto-static benchmark problem with the aim to illustrate their results. In more detail, in [76], the ultimate objective is to develop a virtual sensor system, which refers to a computational method capable of assisting in both the reconstruction of a current based on a set of measurements and the synthesis of current sources based on a specified field distribution in a region of interest. As a lack of example, in the geophysical sector, the same concept can be applied for estimating the physical properties of the ground within the Earth’s interior based on observations gathered either at or above the Earth’s surface by electromagnetic sensors [77].

In [78], a solution to inverse problems and electromagnetic field reconstruction is adopted. It involves a deep learning methodology that uses a conditional variational autoencoder (CVAE) and a convolutional neural network (CNN). The approach consists of two main steps: Initially, given the static magnetic field distribution in a specific subdomain, the entire domain’s magnetic field distribution (field reconstruction problem) is inferred. Subsequently, with knowledge of the magnetic field distribution across the entire domain, a CNN regression model is utilized to identify the geometric properties of the source (current or source identification problem).

In [79], an inverse metamaterial design from an EM near field is demonstrated in optical applications and for sub-wavelength structure. The authors compare the forward solution obtained with topological optimization using a multi-task neural network and the inverse solution of a metamaterial design obtained using the EM near field maps and the inverse EM solution. The authors suggested that their approach was not primarily influenced by the device type, operating frequency range, or design geometry. As a result, it is feasible to use it for the analysis and design of various electromagnetic structures.

Despite this, the specific application of DL for solving inverse problems in EM remains an open challenge, with only a limited number of contributions explicitly investigating this aspect.

## 5. Machine Learning for RF Coil Design Procedure

Machine learning has dominated research in computational MRI during the last few years, but it has also highlighted new issues, such as the application of these methods to the design of MRI coils.

RF coils are usually wire structures, and the numerical method represents the most suitable tool for an accurate and efficient analysis, for example, of the magnetic field inside the coil, even for complex geometries. Many characteristics need to be taken into account when designing RF coils: shape for specific regions of the human body, coil coupling, coil sensitivity, SNR, patient comfort, parallel imaging factors, and image acquisition technique.

Genetic algorithms (GA) may be able to optimize these parameters, since they are more efficient in exploring large search solutions relatively quickly and are very flexible compared to coils designed using iterative trial and error methods.

In this paper, we reviewed techniques based on a genetic algorithm and method of moments (MoM) applied for the design of MRI transmitter/receiver radiofrequency coils. RF coils are usually wire structures. An MoM numerical code represents the most suitable tool for an accurate and efficient analysis of the magnetic field inside the coil, even for complex geometries. Additionally, the GA technique can provide optimum coil parameters, including, for example, the number of coils in the array, the specific size and position of each element for imaging of a specific part of the anatomy, and the signal-to-noise ratio.

D. Yau et al. [80] explored the use of these methods to design an asymmetric RF coil that is useful for organ-specific designs that require flexible geometric structures.

The first step described in the paper is to apply the GA/MoM method to obtain a quasi-static solution for the optimization of an asymmetric RF coil operating at 190 MHz. In particular, the combined method is implemented to search for a time-harmonic solution for the values and positions of the capacitors that produces the required current density and, therefore, field homogeneity. The authors, using a simple coil design (two symmetrical single-loop contours), demonstrate that the genetic algorithm is versatile, with minimal limitations imposed by the geometrical and physical conditions of the structure, and could be a step forward for developing new asymmetric RF coil structures with unusual requirements.

The same approach to determining the geometry of the coil by imposing the homogeneity of the RF magnetic field amplitude in a volume coil was also proposed by Rogovich et al. [81]. The author began from a standard GA procedure, which predicted that the chromosomes of the chosen population of solutions would be composed by a string of genes. They associated a chromosome with a specific volume coil geometry (which cylindrical shape), which could have been a fictitious surface corresponding to a proper projection depending on the shape of the surface. The obtained coil was analyzed through the MoM method to obtain the magnetic field distribution in the region of interest inside the volume coil. The numerical results which they obtained using both the MoM and GA methods demonstrate that the procedure can be applied to non-cylindrical coils.

Hadley et al. [82] used GA to optimize single- and dual-array RF coils for the imaging of vascular structures. They developed a GA tool, by modifying the GA standard method, that could be used for finding the coil geometries and positions that yielded the highest relative SNR for a specific part of the anatomy. The sample noise and signal sensitivity profiles of the coil elements were simulated using the quasi-static equation, taking into account the coil-to-sample-coil-to-coil interactions. The relative signal-to-noise ratio in the structure of interest was used as the cost function for the GA optimization. The simulations reported in the results for only two kinds of coil can be extended to multi-element arrays.

GA algorithms were also applied by Nadig [83] to optimize the magnetic field homogeneity of birdcage coils used in magnetic resonance spectroscopy, which require better SNR for the accurate measurement of the metabolite concentrations. To improve the homogeneity of the magnetic field, they optimized the physical dimensions of the coil, combining the features of the GA and FDTD simulations.

The results showed that GA may be able to generate coils that are more efficient than coils designed by iterative trial and error methods because of the ability of the genetic algorithm to explore a large solution relatively quickly.

Recently, deep learning methods have emerged as powerful tools for the reconstruction of MR images.

These methods can be split into two classes: physics-driven or data-driven.

In particular, physics-driven deep learning methods have not only emerged as powerful tools for the reconstruction of magnetic resonance images, but could also be very promising for an accurate estimation of the receiver coil sensitivity maps. Many methods have been used in the last few years to estimate the receiver coil sensitivity maps. Arshad [84] proposed a trained neural network for receiver coil sensitivity map estimation of a scanner with a different magnetic field strength. They used the concept of fine-tuning for the receiver coil sensitivity map estimation of a 3T scanner starting from the receiver coil sensitivity maps of a 1.5 T scanner, considering the receiver arrays with the same number of receiver coil elements in the source and target domain datasets. The deep learning algorithm results were robust in terms of the estimation of coil sensitivity map.

Electromagnetically trained artificial neural networks were also used by Hui [85] for accurately modeling high-temperature super-conducting (HTS) RF coils. The proposed model solved an inverse problems algorithm starting from a resonant frequency input to derive other properties of the RF coil, with the advantage of an easy output; it was also quick and more interactive then the “moment method”. Furthermore, the simulation reports also show excellent agreement with the experimental measurements.

## 6. Conclusions

In this review, we summarize the state of the art related to the use of ML algorithms for the design and simulation of RF coils for MRI applications. Beginning with a discussion on how such techniques can be employed in MRI, we underline that the development of ML-based coil simulation and design processes can be a fundamental issue that improves the image quality of specific tissues/organs and will become more common in the future by permitting the optimization of different interesting coil design characteristics. The major current limitation in the use of supervised ML for RF coil design is the need for a massive amount of labeled data (i.e., coil geometry and generated EM field pairs) and the time required to effectively train a generalizable model. Producing this kind of data for non-trivial coil geometries is challenging due to the high computational load of EM simulations. The use of physics-informed models and high-performance computing clusters would allow the computational time required for generating representative training data to be shortened.

We believe that this paper contains information that will be useful for researchers involved in the design of MRI coil design.

## Data Availability

Not applicable.
